# Architecture of *Burkholderia cepacia *complex σ^70 ^gene family: evidence of alternative primary and clade-specific factors, and genomic instability

**DOI:** 10.1186/1471-2164-8-308

**Published:** 2007-09-04

**Authors:** Aymeric Menard, Paulina Estrada de los Santos, Arnault Graindorge, Benoit Cournoyer

**Affiliations:** 1 Université de Lyon, Lyon, France; 2 Research group on «Bacterial Opportunistic Pathogens and Environment», UMR5557 Ecologie Microbienne, Université Lyon 1, CNRS, and Ecole Nationale Vétérinaire de Lyon, France; 3 UMR CNRS 5557 Ecologie Microbienne, Mendel Bldg., 5^th ^floor, Université Lyon 1, 69622 Villeurbanne Cedex, France

## Abstract

**Background:**

The *Burkholderia cepacia *complex (Bcc) groups bacterial species with beneficial properties that can improve crop yields or remediate polluted sites but can also lead to dramatic human clinical outcomes among cystic fibrosis (CF) or immuno-compromised individuals. Genome-wide regulatory processes of gene expression could explain parts of this bacterial duality. Transcriptional σ^70 ^factors are components of these processes. They allow the reversible binding of the DNA-dependent RNA polymerase to form the holoenzyme that will lead to mRNA synthesis from a DNA promoter region. Bcc genome-wide analyses were performed to investigate the major evolutionary trends taking place in the σ^70 ^family of these bacteria.

**Results:**

Twenty σ^70 ^paralogous genes were detected in the *Burkholderia cenocepacia *strain J2315 (*Bcen*-J2315) genome, of which 14 were of the ECF (extracytoplasmic function) group. Non-ECF paralogs were related to primary (*rpoD*), alternative primary, stationary phase (*rpoS*), flagellin biosynthesis (*fliA*), and heat shock (*rpoH*) factors. The number of σ^70 ^genetic determinants among this genome was of 2,86 per Mb. This number is lower than the one of *Pseudomonas aeruginosa*, a species found in similar habitats including CF lungs. These two bacterial groups showed strikingly different σ^70 ^family architectures, with only three ECF paralogs in common (*fecI*-like, *pvdS *and *algU*). *Bcen*-J2315 σ^70 ^paralogs showed clade-specific distributions. Some paralogs appeared limited to the ET12 epidemic clone (*ecfA2*), particular Bcc species (*sigI*), the *Burkholderia *genus (*ecfJ*, *ecfF*, and *sigJ*), certain proteobacterial groups (*ecfA1*, *ecfC*, *ecfD*, *ecfE*, *ecfG*, *ecfL*, *ecfM *and *rpoS*), or were broadly distributed in the eubacteria (*ecfI*, *ecfK*, *ecfH*, *ecfB*, and *rpoD*-, *rpoH*-, *fliA*-like genes). Genomic instability of this gene family was driven by chromosomal inversion (*ecfA2*), recent duplication events (*ecfA *and *RpoD*), localized (*ecfG*) and large scale deletions (*sigI*, *sigJ*, *ecfC*, *ecfH*, and *ecfK*), and a phage integration event (*ecfE*).

**Conclusion:**

The Bcc σ^70 ^gene family was found to be under strong selective pressures that could lead to acquisition/deletion, and duplication events modifying its architecture. Comparative analysis of Bcc and *Pseudomonas aeruginosa *σ^70 ^gene families revealed distinct evolutionary strategies, with the Bcc having selected several alternative primary factors, something not recorded among *P. aeruginosa *and only previously reported to occur among the actinobacteria.

## Background

*Burkholderia cepacia *complex (Bcc) bacterial species are β-proteobacteria that can be found in various environments including freshwater and plant rhizosphere. They are also the etiological agents of several human infections. This bacterial complex includes several closely related species or genomovars of *Burkholderia *that can be isolated from cystic fibrosis (CF) patients. These species can also be found in nosocomial infections. *B. cenocepacia *(*Bcen*) is a species of the Bcc that can lead to the "*cepacia *syndrome", a dramatic necrotizing pneumonia (for review see [1]). Three major *B. cenocepacia *epidemic clones have been described around the world: ET12, PHDC and Midwest [2-4]. Three strains of these clonal complexes (one of ET12 and two of PHDC) were sequenced by the Sanger Institute and the Joint Genome Institute, and are available on their respective web site. Several other Bcc and *Burkholderia *strains were sequenced or are in progress of getting sequenced (for a list see [5]).

The ability at colonizing several biotopes is a character observed in the Bcc but also among other bacterial species like *Pseudomonas aeruginosa *of the γ-proteobacteria. This versatility has been linked to an important diversity of regulatory processes including those of the σ^70 ^gene family [6]. Transcriptional σ^70 ^factors can bind reversibly to eubacterial DNA-dependent RNA polymerase (RNAP) to form the holoenzyme, and enable its promoter-specific attachment. This holoenzyme can drive synthesis of mRNA (but the process could be regulated by several transcriptional repressors [7] and activators [8]). Through evolution, selective pressures seem to have favoured the emergence of several paralogous lineages of σ^70^, and these factors became a central component of bacterial adaptability to changing environments including host colonization. This resulted in the observation of some sort of relation between the lifestyle of bacteria and the number of genes encoding σ^70 ^factors in their genome. For example, 65 σ^70 ^gene sequences were detected among the genome of *Streptomyces coelicolor *[9] which is ubiquitous and morphologically complex but only one in *Mycoplasma *spp. which are host- and disease-specific [10,11]. Among *Pseudomonas aeruginosa*, a species sharing several niches with the Bcc, 23 σ^70 ^gene sequences have been reported [6]. Generally speaking, σ^70 ^factors can be divided into four groups according to sequence similarities and promoter-recognition specificities [12,13]: (1) the essential primary sigma factors, involved mainly in exponential growth, and the alternative actinomycetal sigma factors; (2) the stationary phase factors; (3) the factors involved in sporangium development, flagellin synthesis and heat shock response, and (4) the extracytoplasmic function sigma factors (termed ECF). All these factors are in competition for the same RNAP core enzyme [14]. Genes under the control of a same sigma factor are termed to be part of a same sigmulon.

Here, we report the first global analysis of the σ^70 ^gene family among the *Burkholderia cepacia *complex. The first fully sequenced Bcc genome of *B. cenocepacia *(strain J2315 of the ET12 epidemic clone) was used as the reference sequence for this study.

## Results and Discussion

### Detection and number of σ^70 ^gene sequences in Bcen-J2315 genome

σ ^70 ^TBLASTN searches of *B. cenocepacia *strain J2315 (*Bcen*-J2315) genome sequence were performed. First, searches making use of ten σ^70 ^factors of the main groups of this family were carried out. Then, a second round of TBLASTN searches using all *B. cenocepacia *factors detected in the initial screening was done. This led to the identification of twenty σ^70 ^gene sequences, thirteen on chromosome 1, seven on chromosome 2, and none on chromosome 3 (Table 1). All genomic regions harbouring σ^70 ^coding sequences (CDS) were analysed by the testcode programme, to identify their corresponding ORF. In the case of the presence of multiple initiation codons, the ORF showing the longest CDS was selected. All the defined σ^70 ^gene sequences and their position on the *Bcen*-J2315 genome are listed in Table 1. Given the genome size of *Bcen*-J2315 (8,056 Mb), an average of 2,86 σ^70 ^gene sequences per Mb was computed. This number of sigma genes is slightly lower than the number reported in *P. aeruginosa *(23 σ^70 ^in strain PA01, genome size of 6,26 Mb) for which an average of 3,67 σ^70 ^genes per Mb was calculated. It is also lower than the one of the genome of *Streptomyces coelicolor *strain A3(2) (8,67 Mb) which has a similar genome size. However, *S. coelicolor *has a very distinct morphology (being hyphal and producing spores) and ecology. This simplistic analysis suggests that bacteria like the Bcc and *P. aeruginosa*, which are sharing several habitats, have similar number of σ^70 ^gene sequences. However, the composition of these families was found to be quite different (as described in the next sections).

**Table 1:** Distribution of *B. cenocepacia *J2315 strain σ^70 ^factors among other *Burkholderia *genome sequences and *P. aeruginosa*.

				*Bcc*					*pseudomallei *lineage				
							
*Bcen *σ ^70 ^factor	associated name	cds position in J2315 genome	locus tag	*B. cenocepacia*	*B. vietnamiensis*	*B. sp. 383*	*B. ambifaria*	*B. dolosa*	*B. mallei*	*B. pseudomallei*	*B. thailandensis*	*B. xenovorans*	*P. aeruginosa*
*Bcen*-SigE	RpoD	chr.2 1009395*-1011812	BCAM0918	4/4	1/1	1/1	1/1	1/1	4/4	6/6	1/1	1/1	2/2
*Bcen*-SigI		chr.2 2666931-2668817	BCAM2371	4/4	1/1	0/1	1/1	1/1	0/4	0/6	0/1	0/1	0/2
*Bcen*-SigJ		chr.2 1383611-1382085	BCAM1259	3/4	1/1	1/1	1/1	1/1	4/4	6/6	1/1	0/1	0/2
*Bcen*-SigL	RpoH-like	chr.1 2088369-2089457	BCAL1892	4/4	1/1	1/1	1/1	1/1	4/4	6/6	1/1	1/1	2/2
*Bcen*-SigM	FliA-like	chr.1 860723-859788	BCAL0787	4/4	1/1	1/1	1/1	1/1	4/4	6/6	1/1	1/1	2/2
*Bcen*-SigK	RpoS	chr.1 168141-168872	BCAL0144	4/4	1/1	1/1	1/1	1/1	4/4	6/6	1/1	1/1	2/2
*Bcen*-EcfA1	RpoE	chr.1 1085335-1085934	BCAL0998	4/4	1/1	1/1	1/1	1/1	4/4	6/6	1/1	1/1	2/2
*Bcen*-EcfA2	RpoE	chr.1 153677-153078	BCAL2872	1/4	0/1	0/1	0/1	0/1	0/4	0/6	0/1	0/1	0/2
*Bcen*-EcfB	PrtI-like	chr.1 3817661-3818182	BCAL3478	4/4	1/1	1/1	1/1	1/1	0/4	6/6	1/1	0/1	0/2
*Bcen*-EcfC	FecI-like	chr.1 1506330-1506836	BCAL1369	4/4	0/1	1/1	0/1	0/1	4/4	6/6	1/1	0/1	2/2
*Bcen*-EcfD		chr.1 2615164-2615727	BCAL2360	4/4	1/1	1/1	1/1	1/1	4/4	6/6	1/1	1/1	0/2
*Bcen*-EcfE		chr.1 2728250-2727696	BCAL2462	4/4	0/1	1/1	0/1	1/1	0/4	0/6	0/1	1/1	0/2
*Bcen*-EcfF		chr.1 3441702-3441187	BCAL3151	4/4	1/1	1/1	1/1	1/1	4/4	6/6	1/1	1/1	0/2
*Bcen*-EcfG		chr.1 2146519*-2145818	BCAL1947	4/4	0/1	1/1	1/1	1/1	4/4	6/6	1/1	1/1	0/2
*Bcen*-EcfH		chr.2 1846346-1845561	BCAM1661	4/4	0/1	1/1	1/1	0/1	0/4	0/6	0/1	1/1	0/2
*Bcen*-EcfI	OrbS	chr.1 1846454-1847122	BCAL1688	4/4	1/1	1/1	1/1	1/1	4/4	6/6	1/1	1/1	2/2^•^
*Bcen*-EcfJ		chr.2 230-829	BCAM0001	4/4	1/1	1/1	1/1	1/1	4/4	6/6	1/1	0/1	0/2
*Bcen*-EcfK		chr.2 3109088-3109981	BCAM2748	4/4	0/1	1/1	0/1	1/1	4/4	6/6	1/1	0/1	0/2
*Bcen*-EcfL		chr.2 934738-936006	BCAM0849	4/4	1/1	1/1	1/1	1/1	4/4	6/6	1/1	1/1	0/2
*Bcen*-EcfM		chr.1 3824313-3823432	BCAL3486	4/4	1/1	1/1	1/1	1/1	0/4	0/6	0/1	0/1	0/2

TBLASTN searches of *Bce*-J2315 σ factors were performed on fully sequenced *Burkholderia *genomes. In accordance with phylogenetic inferences shown in Fig. 3 and 4, TBLASTN were considered to reveal homologs or orthologs of a same σ^70 ^lineage when showing identity values above 50% and similarity higher than 63%, and when being part of a phylogenetic cluster supported by high bootstrap values. • *P. aeruginosa *OrbS homologs, named PvdS, showed 38% identity and 56% similarity with *Bcen*-J2315 homolog. Sequences were retrieved from [51-54]. Genomes of the following strains were analysed: *B. cenocepacia*, strains J2315, AU1054, HI2424, PC184 (negative for *Bcen*-SigJ); *B. vietnamiensis*, G4; *Burkholderia sp.*, 383 ; *B. ambifaria*, AMMD, *B. dolosa*, AU0158; *B. xenovorans*, LB400; *B. mallei*, ATCC23344, 10229, GB8 horse 4, SAVP1; *B. pseudomallei*, 1710a, 1710b, S13, 1655, 668, K96243; *B. thailendensis*, E264; *P. aeruginosa*, PAO1, PA14. * indicates an ORF different from the one of the annotated genome.  Distribution limited to *Burkholderia *genomes, see Fig. 4.

### Classification of σ^70 ^gene sequences of Bcen-J2315 into functional categories

**Figure 1:**
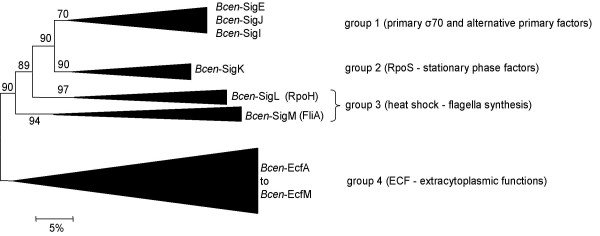
**Allocation of *B. cenocepacia *strain J2315 σ ^70 ^deduced factors into the main σ ^70 ^gene family phylogenetic groups**. All *Bcen*-J2315 deduced amino acids sequences from σ^70 ^coding sequences were added to the frame of the Lavire et al. [13] multiple alignment. A Neighbor-joining phylogenetic tree was computed from this multiple alignment. Distances on this tree are proportional to evolutionary divergences expressed in substitutions per 100 sites. The *Bcen*-J2315 sequences were allocated to one of the following phylogenetic groups: group 1, the essential primary factors, and alternative primary factors of the actinobacteria; group 2, stationary phase factors of the RpoS type; group 3, factors involved in heat shock response, flagella synthesis or cellular differentiation, and group 4, factors involved in extracytoplasmic functions (ECF).

Translated amino acid (AA) sequences from *Bcen*-J2315 σ^70 ^coding sequences were positioned among the main eubacterial σ^70 ^lineages, using a decisional σ^70 ^Neighbor-Joining (NJ) phylogenetic tree limited to key sequences (Fig. 1). This phylogenetic tree divides the σ^70 ^gene family into four groups (see [13]): (1) one grouping the primary (mainly RpoD) and alternative primary actinomycetal factors, (2) one grouping the stationary phase factors (mainly named RpoS), (3) one the heat shock (RpoH), flagellin (FliA), and cellular differentiation (WhiG) factors, and (4) one grouping factors involved in extracytoplasmic functions (named ECF factors). All these groups are supported by high bootstrap values. This phylogenetic tree allowed to allocate 14 of the *Bcen*-J2315 σ^70 ^gene sequences into the ECF (see Table 1). The ECF group is often the most important in number, and is the main group involved in bacterial responses toward changing environmental conditions. ECF sequences represent 66% of the σ^70 ^gene sequences of the *Bcen*-J2315 genome, a number which is lower than the one observed in *P. aeruginosa *(82%) [6,15]. Two deduced AA sequences, EcfA1 and EcfA2, are 100% identical in *Bcen*-J2315, and their respective genes are positioned on two identical DNA strands of 40 kb that are diametrically opposed on chromosome 1 (see section on *ecfA *for further details). For the remaining 6 factors, three were allocated to group 1 (SigE, SigI and SigJ), one to group 2 (SigK), and two to group 3 (SigL and SigM).

**Figure 2:**
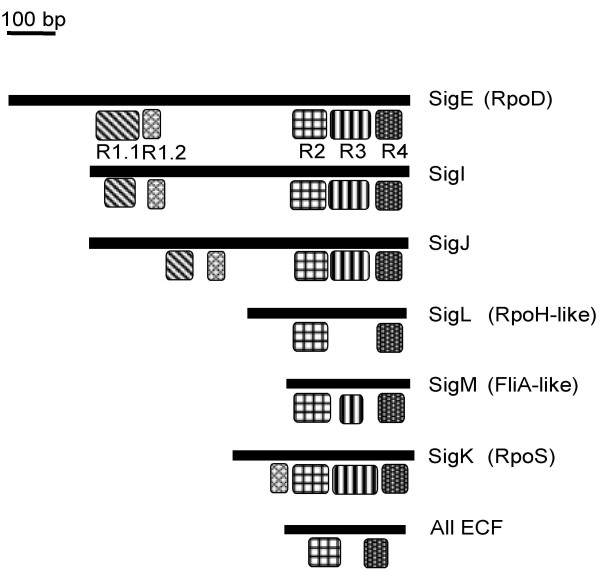
**Conserved regions among *B. cenocepacia *strain J2315 σ ^70 ^factors**. These regions were detected by CDD (Conserved Domain Detection [42]). R1.1: region 1.1 can modulate or prevent DNA binding by regions 2 and 4 when the sigma is unbound by the core RNA polymerase; R2: region 2 contains both the -10 promoter recognition helix and the primary core RNA polymerase binding domain. R3: region 3 is primarily involved in binding to the core RNA polymerase. R4: region 4 is involved in binding to the -35 promoter region.

*Bcen*-J2315 deduced σ^70 ^factors conserved domains were revealed by CDD analyses (Fig. 2). Four conserved domains (regions 1 to 4) were described [12] among the σ^70 ^family, with region 1 being sub-divided into R1_1 and R1_2. *Bcen*-J2315 SigE, I and J contain all four regions. Regions 2 and 4 were identified in *Bcen*-J2315 deduced SigL, SigM and SigK sequences but only a full region 3 was found in SigM and SigK. Sub region 1_1 was detected in SigK. Only regions 2 and 4 were detected among the ECF factors.

### Molecular phylogeny of Bcen-J2315 primary, stationary, and stress response/cellular differentiation/flagellin synthesis σ^70 ^gene sequences

The NJ tree of Fig. 1 could not be used to asses accurately the phylogenetic relationships among each of the main σ^70 ^phylogenetic groups. The phylogenetic resolution of the σ^70 ^family appeared to be more robustly determined by splitting the dataset into two subsets corresponding to ECF and non-ECF (primary, stationary, and stress response/cellular differentiation) deduced σ^70 ^AA sequences.

**Figure 3:**
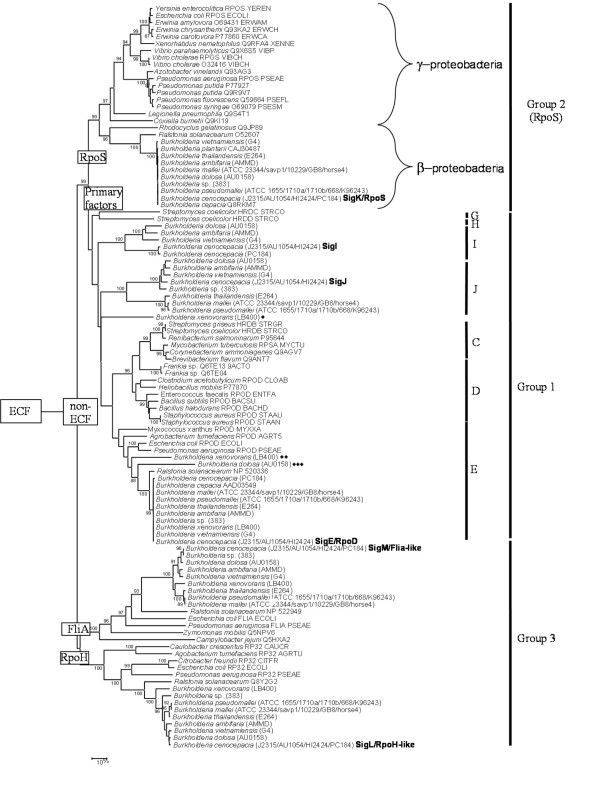
**Neighbor-Joining phylogenic tree of non-ECF σ ^70 ^factors**. The σ^70 ^sequences were from [13] or were retrieved from sequenced *Burkholderia *genomes or the GenBank database. Within a single species only one ortholog per sigma lineage was kept. A total of 178 sites were analyzed (with global removal of gap-containing sites). Distances are proportional to evolutionary divergences expressed in substitutions per 100 sites. Bootstrap values higher than 85% are given. Sub-groups are defined in the text. (*) *B. xenovorans *LB400 alternative primary factor. (**) additional copy of *rpoD *found in *B. xenovorans *LB400 genome. (***) Deduced *B. dolosa *AU0158 RpoD sequence that probably contains a frameshift due to a DNA sequencing mistake. Main phylogenetic groups of non-ECF factors are indicated on the figure: group 1, primary and alternative primary factors; group 2, stationary phase factors; and group 3, heat shock (RpoH), flagellin (FliA), and cellular differentiation factors

Reliable phylogenetic positioning of *Bcen*-J2315 deduced σ^70 ^factors was performed by first extracting all AA sequences in the databases showing significant similarities with these factors. These sequences were then added to the multiple alignment obtained by Lavire et al. [13], and a NJ phylogenetic tree was inferred (Fig. 3). Names of lineages used in Lavire et al. [13] were kept. The NJ tree of non-ECF AA sequences is divided into three major clusters of paralogs, matching the non-ECF categories described in the above section (groups 1, 2 and 3). All these paralogous σ^70 ^sequences appear to have emerged early in the evolution of the eubacteria. The SigE/RpoD (group 1), SigM/FliA and SigL/RpoH homologs (group 3) were detected among at least the α-, β-, and γ-proteobacteria, suggesting an emergence prior to the diversification of these bacterial groups. However, the group 2 factors (RpoS) were found restricted to β-proteobacteria and γ-proteobacteria.

#### - the primary and alternative primary σ^70 ^factors phylogenetic radiation (group 1)

In the NJ tree of non-ECF σ^70 ^factors (Fig. 3), the group of primary and alternative primary factors was found divided into 7 distinct and significant lineages: C, D and E corresponding to the essential eubacterial primary factors, G and H being representatives of the alternative primary factors lineages of the actinobacteria, and I and J being two novel lineages. *Bcen*-J2315 SigE and its *Burkholderia *orthologs are found to be part of cluster E, the RpoD lineage of essential sigma factors of the proteobacteria, and were thus renamed RpoD. A *B. dolosa *strain AU0158 RpoD-like sequence appeared distantly related to other *Burkholderia *deduced RpoD but a closer look at its gene structure showed a frameshift probably due to a sequencing mistake. An additional copy of the *rpoD *gene was detected in the *B. xenovorans *genome (Fig. 3). This copy shows a deduced AA sequence significantly different from the ones of the *sigE/rpoD Burkholderia *orthologs, suggesting either acquisition by an horizontal transfer event or an ancient duplication event prior to the diversification of the β-proteobacteria.

*Bcen*-J2315 SigI and SigJ deduced sequences and their orthologs revealed two novel lineages of group 1 factors which are restricted to the *Burkholderia *genomes (Fig. 3), and were thus termed alternative *Burkholderia *primary factors. The *sigJ *coding sequence was not detected in *B. xenovorans *and *B. cenocepacia *PC184 genomes (Table 2 and see section on genomic rearrangements). However, *sigJ *DNA probings of Bcc genomic DNA blots showed a broad distribution among the Bcc (including *B. cepacia, B. cenocepacia*, *B. stabilis*, *B. dolosa, B. vietnamiensis, B. multivorans *and *B. pyrrocinia*) (Table 2). *sigI *was not detected in the genomes of the Bcc strain 383, of *B. xenovorans*, and of *B. pseudomallei *and its closely related genomes (see section on genomic rearrangements). Nevertheless, Bcc DNA blot analyses using *sigI *DNA probes showed this gene largely distributed in the Bcc (Table 2). In *B. xenovorans *LB400 genome, *sigI *and *sigJ *were not detected but another lineage of alternative *Burkholderia *primary factors was found. The presence of coding sequences of such factors can thus be considered a common feature of *Burkholderia *genomes. Alternative primary factors were never detected in *P. aeruginosa*. In fact, this is the first report demonstrating the presence of alternative primary factors among bacteria outside the actinomycetes. The function of these alternative primary factors is unknown but they possess all the domains found among the RpoD factors i. e. -10 and -35 promoter recognition domains, RNA polymerase binding domains, etc (Fig. 2). These factors could be replacing the primary one under growth conditions inhibiting its expression or functioning (potentially certain antibiotics).

**Table 2:** *Burkholderia cepacia *complex strains used in this study, and summary of DNA blot analyses of these strains using *ecfB*, *ecfD*, *ecfE*, *sigI*, and *sigJ *DNA probes.

Strains	References	*ecfB*	*ecfD*	*ecfE*	*sigI*	*sigJ*
*B. cepacia*						
LMG 1222	[55]	+	+	+	+	+
AU0113	[56]	+	+	+	+	+
LMG 2161	[55]	+	+	+	+	+
*B. stabilis*						
LMG 14294	[57]	+	+	+	+	+
AU0244	[58]	+	+	+	+	+
*B. cenocepacia*						
J2315	[59]	+	+	+	+	+
H111	[60]	+	+	+	+	+
IST404	[61]	+	+	+	+	+
BCC020	this study	+	+	+	+	+
LMG 18828	[59]	+	+	+	+	+
LMG 18830	[59]	+	+	+	+	+
BCC021	this study	+	+	+	+	+
LMG 21440	[59]	+	+	+	+	+
LMG 18829	[59]	+	+	+	+	+
*B. multivorans*						
LMG 13010	[62]	+	+	-	-(+)*	+
LMG 16660	[62]	+	+	-	-(+)*	+
LMG 18822	[62]	+	+	-	-(+)*	+
C1962	[62]	+	+	-	-(+)*	+
LMG 16665	[62]	+	+	-	-(+)*	+
*B. vietnamiensis*						
LMG 10929	[63]	+	+	-	-(+)*	+
ATCC 53617	[63]	+	+	-	-(+)*	+
CL	[64]	+	+	-	-(+)*	+
LMG 16232	[63]	+	+	-	-(+)*	+
LMG 16228	[63]	+	+	-	-(+)*	+
LMG 17830	[63]	+	+	-	-(+)*	+
LMG 10823	[63]	+	+	-	-(+)*	+
*B. dolosa*						
LMG 18941	[65]	+	+	+	-(+)*	+
*B. pyrrocinia*						
LMG 14191	[66]	+	+	+	+	+

^32^P-random labelling of DNA probes were done as described in the Methods section. See Table 3 for position of the PCR products used for the synthesis of DNA probes. + : positive hybridization signal, - : no hybridization signal (<72% identity). * : hydridization signals varied according to the *sigI *DNA probe used. (+): these positive signals were only obtained with the *Bvi-sigI *probe.

#### - the stationary phase σ^70 ^factors phylogenetic radiation (group 2)

The group 2 phylogenetic radiation is divided into two clusters (supported by 96% and 97% of the bootstrap replicates, respectively) which match the diversification of the proteobacteria into the β and γ subgroups (Fig. 3). *Bcen*-J2315 *sigK *deduced amino acids sequence is clustered with the β-proteobacteria RpoS deduced sequences (and was thus renamed *rpoS*). In *B. cepacia *and *B. pseudomallei*, RpoS was shown to be implicated in oxidative stress response, to be induced by heat shock treatments, and upon carbon starvation [16,17]. No RpoS-like coding sequence was detected in any α-proteobacterial or other eubacterial genomes. Among the actinomycetes, the stationary phase factors are known to be part of a fifth group of sigma factors which was not considered in this study i.e. the lineage of actinomycetal stress response and stationary phase factors (including sequences like *S. coelicolor *RpoX, *Mycobacterium tuberculosis *SigF, etc) [18,19]. This group is not closely related to group 2. Misleading automatic annotations of several RpoS gene sequences were observed in *Borrelia*, *Bacteroides*, *Blastopirellula*, *Rhodopirellula*, *Moorella *or *Aquifex *genome; their deduced factors did not group in the RpoS cluster (data not shown). To clarify this situation, we suggest the use of the name *rpoS *only for sequences of the group 2 σ^70 ^factors from the β- and γ-proteobacteria. The restricted distribution of RpoS gene sequences and their close phylogenetic proximity with Group 1 sequences (primary and alternative primary factors) suggest a likely emergence of *rpoS *from an ancestral group 1 sequence. A duplication of the highly conserved eubacterial *rpoD *gene probably led to this paralogous group. Alternative primary factors described in the above section might have evolved from similar but more recent duplication events.

The emergence of *rpoS *can be considered a key event in the evolution of the eubacteria. Molecular phylogenies of RpoD (group 1), and FliA-like and RpoH-like sequences (group 3) (Fig. 3) suggest an early diversification of the α-proteobacterial sequences from those of the β- and γ-proteobacterial sequences. This can also be inferred from other datasets e.g. *rrs *and concatenation of essential proteins [20]. The emergence of *rpoS *matches the separation of the α-proteobacteria (no *rpoS *– ancestral state) from the β- and γ-proteobacteria (harbouring *rpoS*).

#### - the stress response/cellular differentiation/flagellin synthesis σ^70 ^factors phylogenetic radiation (group 3)

Group 3 is polyphyletic and divided into two groups, one corresponding to FliA-like sequences, and one corresponding to RpoH-like ones (supported by 100% of the bootstrap replicates). *Bcen*-J2315 *sigM *deduced AA sequence belongs to the FliA subgroup, whereas *Bcen*-J2315 *sigL *deduced AA sequence groups with the RpoH-like sequences (Fig. 3). A *sigM *(*fliA*) homolog was confirmed to be involved in motility among the *Burkholderia *[21].

### Molecular phylogeny of Bcen-J2315 extracytoplasmic functions σ^70 ^gene sequences (group 4)

**Figure 4:**
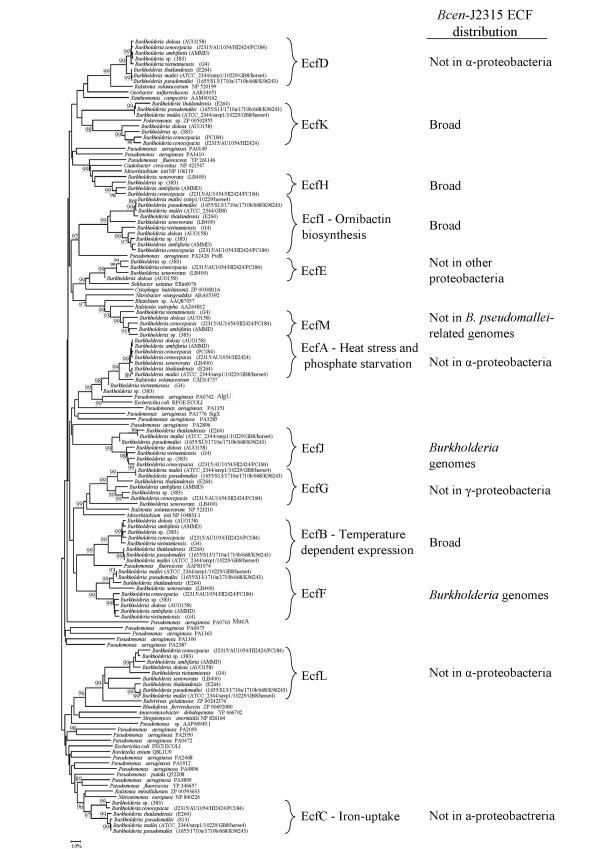
**Neighbor-Joining phylogenic tree of ECF σ ^70 ^factors**. The sequences were retrieved from sequenced *Burkholderia *genomes or the GenBank database. Within one species, when sequences were orthologs, only one sequence per sigma factor was kept. A total of 746 sites were analyzed (with pairwise deletion of gap-containing sites). Distances are proportional to evolutionary divergences expressed in substitutions per 100 sites. Bootstrap values higher than 85% are given. Distribution among other proteobacterial groups is indicated. Likely role of the identified ECF factors is indicated.

Phylogenetic relationships among the ECF cluster are presented in Fig. 4. All database sequences closely related to *Bcen*-J2315 putative ECF were considered in this analysis. A first round of phylogenetic analysis allowed the identification of all database factors related to *Bcen*-J2315 ECFs. A second round of analyses was then performed by limiting the dataset to a set of selected sequences representative of the main phylogenetic trends observed. Fig. 4 shows the best NJ tree obtained. Its structure is not supported by high bootstrap values but all sub-groups of ECFs are supported by high ones. EcfA, EcfC and EcfI deduced AA sequences are the only *Bcen*-J2315 ECF factors having homologs in the *P. aeruginosa *genomes.

#### - *ecfA*: evidence of an ET12 clone-specific duplication event

EcfA-like deduced sequences are found in several proteobacterial genomes but could not be detected in the ones of α-proteobacteria (Fig. 4). Surprisingly, *B. vietnamiensis *and *Burkholderia *sp. 383 deduced EcfA sequences were found to group apart from other EcfA-like sequences belonging to the *Burkholderia *(Fig. 4); making these sequences closer relatives of a *Ralstonia *homologous sequence. *ecfA *encodes a factor highly similar to the well-characterized AlgU factor of *P. aeruginosa *which is involved in alginate biosynthesis [22]. However, the *B. cepacia *homolog of EcfA was recently shown not to be involved in exopolysaccharide biosynthesis and onion pathogenicity. It was found involved in the adaptation to heat stress and phosphate starvation [23]. Interestingly, the *ecfA *gene sequence was found in two copies in the *Bcen*-J2315 genome. These two copies are harboured by identical 40 kb DNA fragments delimited, on one hand, by an IS element.

**Figure 6:**
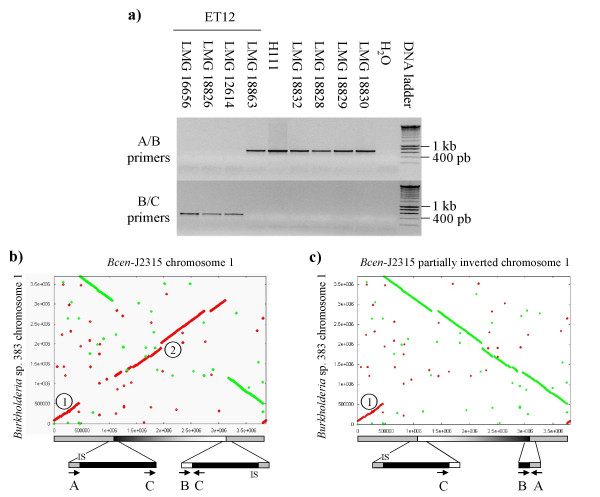
**The *ecfA *(*rpoE*) gene duplication**. (a) PCR analysis of the duplicated 40 kb *ecfA *region among *Bcc *genomes. Strains used are described in Table 2. PCR primers are presented in Table 3. The DNA size marker is shown. Orientation of the PCR primers is indicated in the b and c sections. B/C primers detect the *Bcen*-J2315 configuration (duplication event)-*ecfA2 *allele, and A/B primers detect the *Burkholderia *sp. 383 configuration (no inversion). (b) and (c) Promer plot comparison of *Burkholderia *sp. 383 chromosome 1 against either (b) *Bcen*-J2315 chromosome 1 or (c) the partially inverted *Bcen*-J2315 chromosome 1.  inverted region #1 an  inverted region #2 of *Bcen*-J2315. ISBcen8 position is indicated. Promer analyses were performed according to [25].

To trace back the *ecfA *duplication event in *B. cenocepacia*, a PCR strategy was built. PCR primers were defined for each hand of the duplicated 40 kb regions containing the *ecfA *copies (Fig. 6a). PCR products from the B and C primers (Table 3), targeting the duplicated region containing the *ecfA2 *allele, were obtained for all but the LMG 18863 strain of the ET12 clonal complex; a strain confirmed to be a member of the ET12 clone by PCR screening of the BCESM and *cblA *loci (data not shown, see [24]). PCR products amplified with the A and B primers (Table 3), targeting the end of the DNA region affected by the *ecfA *duplication and its linked chromosomal inversion, were obtained for this latter ET12 strain and the non-ET12 *B. cenocepacia *isolates. *B. cenocepacia *PHDC strains (AU1054 and HI2424) and *B. cenocepacia *PC184 genomes harboured only the ecfA1 allele. The ET12 LMG 18863 strain, which was not found to harbour the 40 kb *ecfA*-duplicated region, could have either reverted to its previous genetic organization or could be a more ancestral representative of the ET12 clonal complex. These data suggest that duplication of the *ecfA *40 kb locus could have contributed to the radiation of the ET12 complex.

**Table 3:** PCR primers used in this study.

DNA targets	PCR primer code	DNA sequence	position on *Bcen*-J2315 genome	annealing temperature
*ecfA1 *region	A	CACGGTCATCACGCTCGC	Chr1 3124945-3124962	55°C
*ecfA1 *and *ecfA2 *region	B	TTCGGTGCGATAACCAGC	Chr1 1056358-1056374	55°C
*ecfA2 *region	C	CGGGCGCAAGAACATACA	Chr1 1056771-1056789	55°C
*ecfB*	ecfB-F	CGCTTCGCGCTCTGGCTG	Chr1 3817718-3817735	58°C
*ecfB*	ecfB-R	TCAGCCGCAAGGAAGGAGC	Chr1 3818171-3818153	58°C
*ecfD*	ecfD-F	CGCAACTGCGGCGCCCG	Chr1 2115044-2115060	55°C
*ecfD*	ecfD-R	CGAGCGTTTCGAGCGGGTC	Chr1 2615449-2615467	55°C
*ecfE*	ecfE-F	GTGAACGAACGAGGCTAGG	Chr1 2728191-2728210	55°C
*ecfE*	ecfE-R	CTCCTTGAAGGACTGTCC	Chr1 2727807-2727824	55°C
*sigI*	sigI-F	ATCCCGCTCGATGCCGCGC	Chr2 2667864-2667882	55°C
*sigI*	sigI-R	GAGAATTTGAAGCCGCGCCG	Chr2 2668242-2668261	55°C
*sigI*	sigIv-F	ATCATGCCCGCTGGCTC	Chr3* 1181202-1181218	55°C
*sigI*	sigIv-R	AACAGCCCGATGTTGCCT	Chr3* 1181508-1181491	55°C
*sigJ*	sigJ-F	CATGCGCATGGCACCCGTTC	Chr2 1383013-1383032	55°C
*sigJ*	sigJ-R	GAGAATTTGAAGCCGCGCCG	Chr2 2668242-2668261	55°C

* Position on *B. vietnamiensis *G4 genome

*Bcen*-J2315 chromosome 1, was compared to chromosome 1 of *Burkholderia *sp. 383 using the Promer tool according to [25]. This tool provides large scale DNA sequence analysis. ORFs were automatically detected and similar ones were plotted on Fig. 6b. This analysis detected two inversion events on these chromosomes. To determine the role of *ecfA*-40 kb region in these duplication events, the chromosome 1 region was inverted manually between the two 40 kb duplications and compared with *Burkholderia *sp. 383 chromosome 1 (Fig. 6c). In this way, only the first inversion is detected, indicating that the 40 kb duplication was implicated in the large scale inversion of *Bcen*-J2315 chromosome 1. One border of the 40 kb duplication was found to harbor an Insertion Sequence (IS) named ISBcen8 according to the IS Database [26]. This IS could have played a role in the duplication event that led to the inversion of an important part of chromosome 1 of ET12 strains. This insertion sequence is found (with at least 99% identity) in seven copies on chromosome 1, two copies on chromosome 2, and 3 copies on chromosome 3. The ORF encoding the transposase of this IS was identified with ORF finder and confirmed by GC skew analysis. IS492 from the IS110 family (ORF: 34% identity, 52% similarity) was found to be the closest phyletic neighbour of this IS.

In several species such as *P. aeruginosa*, *Salmonella typhi *or *Bordetella pertussis*, genome rearrangements were found associated with infectious outbreaks [27-29]. Hughes et al. [30] suggested that rearranged genomes could allow to overcome the immune system of the host. The *ecfA *40 kb locus could thus have contributed to the emergence of the hyper-virulent ET12 infra-specific clonal complex by driving a large scale inversion of chromosome 1.

#### - *ecfB*: involvement in temperature-dependent processes

This ECF lineage has a broad distribution (Fig. 4). Related genes could be found in the α and γ-proteobacteria. *ecfB *DNA probings of Bcc genomic DNA blots confirmed its large distribution in the Bcc (Table 2). The deduced EcfB protein is phylogenetically closely related to PrtI which is involved in a temperature dependent regulation of certain proteases [31]. However, no gene at the proximity of *ecfB *was found to encode a protease in *Bcen*-J2315 and other *Burkholderia *genomes. Such a situation was also observed for the PrtI-like locus of the *P. putida *genome [6]. *ecfB *could thus be involved in temperature dependent regulatory processes but seems to have been recruited for the regulation of other protein families than proteases.

#### - *ecfC *and *ecfI*: ECF gene sequences involved in iron-uptake

The ECF NJ tree (Fig. 4) shows *Bcen*-J2315 deduced EcfI sigma factor to group with the PvdS factor from *Pseudomonas aeruginosa*, and the EcfC deduced factor to group with FecI-like factors. EcfI was previously shown to be involved in siderophore (ornibactin) biosynthesis [32] (EcfI being named OrbS in this latter article). Here, it is found to have a broad distribution (Fig. 4). FecI was shown to be involved in the regulation of iron dicitrate uptake in *E. coli *[33]. In other bacterial species, the *fecIRA *system can regulate iron uptake by other siderophores than citrate [34]. Phylogenetic analysis of EcfC/FecI homologs suggests an emergence of this particular sub-group after the diversification of the *α*-proteobacteria (no *fecI *homologs) from the other proteobacteria. Interestingly, FecI-like factors represented 26% of *P. aeruginosa *deduced ECF factors, and 68% of those of *P. putida *[6]. Among *P. aeruginosa *and *P. putida*, these systems were all physically linked to genes involved in the synthesis of outer membrane siderophore receptor proteins. The high numbers of FecI paralogs in these two pseudomonads were considered indicative of a shared ability at colonizing certain niches that require distinct iron uptake systems or particular interactions with siderophores [6]. In the case of the Bcc, a single FecI-like factor was detected. The *ecfC *gene, encoding this factor, was found physically linked to *fecR*- and *fecA*-like gene sequences. FecI can interact with FecR to activate the *fecABCDE *operon involved in the transport of extracellular Fe(III)-citrate complexes to the cytoplasm. FecR interaction is modulated by FecA which will activate the FecIR interactions after perception of Fe(III)-citrate complexes. TBLASTN searches using FecR were performed on selected Bcc genomes to ensure that no FecIRA-like system was missed during our analyses. These searches did not detect any other FecIRA system in *Bcen*-J2315, *B. vietnamiensis*, *B. dolosa*, and *B. ambifaria *genomes. Other TBLASTN searches using HemO, CirA, Has, HxuC, and *P. aeruginosa *SigX, FiuI, PigD, and PupI-homologs were performed to complete this analysis. None of these BLAST searches revealed novel coding sequences involved in haem- or iron-uptake [6] in *Bcen*-J2315.

Interestingly, *B. cenocepacia *was recently shown able to get its iron from ferritin by proteolytic degradation of this iron-binding protein. *B. cenocepacia *is apparently the only known pathogenic bacteria able to use ferritin as an iron source [35]. The iron uptake strategies selected by *P. aeruginosa *and the Bcc are thus quite different; with *P. aeruginosa *showing a greater versatility in ECF regulated processes.

#### - *ecfD*, *ecfE*, *ecfG*, *ecfH*, *ecfL *and *ecfM *loci: unknown physiological implications

The physiological processes involving these regulatory factors remain unknown. However, their distribution and evolutionary history reveal several observations suggesting that they play significant roles.

EcfD is grouping with ECF factors deduced from genomes of other proteobacterial groups (γ and Δ) but not α-proteobacterial ones. *ecfD *DNA probings of Bcc genomic DNA blots showed a large distribution in the Bcc (Table 2). EcfE is not grouping with ECF factors from other proteobacteria but is closely related to σ factors found among *Solibacter usitatus *and *Cytophaga hutchinsonii*. *ecfE *DNA probings gave positive hybridization signals on *B. cepacia, B. cenocepacia*, *B. stabilis*, *B. dolosa *and *B. pyrrocinia *genomic DNA blots (Table 2). However, restricted genomic DNA from all strains of *B. multivorans *and *B. vietnamiensis*, tested in this study, did not yield positive hybridization signals with the *ecfE *probe. Phage DNA might have played a role in the loss of *ecfE *in these bacterial species (see section on genomic rearrangements). Surprisingly, the *ecfE *deduced AA sequence of *B. dolosa *AU0158 was found distantly related to other homologs of the Bcc. EcfG is significantly grouping with σ factors deduced from other β- and α-proteobacterial genomes but not with γ-proteobacterial ones. In this case, a deletion of *ecfG*-like sequences probably occurred early in the evolution of the γ-proteobacteria. Concerning the deduced AA sequences of *ecfK *and *ecfH *coding regions, homologs could be detected in genomes of the three main proteobacterial groups (α,β, and γ). *Bcen*-J2315 EcfH deduced homologs were mainly found in genomes of α-proteobacteria but a sequence was also detected among one γ-proteobacterial genome, the one of *Pseudomonas fluorescens*. *ecfH *was not detected in the *B. vietnamiensis *G4 and *B. ambifaria *AMMD genomes (see section on genomic rearrangements). EcfK homologs were found in other *Burkholderia *genomes and in the one of a species of *Polaromonas*, which is also part of the β-proteobacteria. *ecfK *was not detected in the *B. vietnamiensis *G4 and *B. ambifaria *AMMD genomes (see section on genomic rearragements). EcfL is clustered with ECF without any known function but encoding a TPR (Tetratricopeptide repeat) motif involved in protein-protein interactions. This motif is not found in EcfL and its related *Burkholderia *ECF factors. In fact, deduced EcfL-like sequences retrieved from *B. ambifaria *AMMD, *B. cenocepacia *PC184 and *B. pseudomallei *668 genomes are misleadingly annotated with a TPR repeat motif in the databases. EcfL coding gene sequences are found in the genomes of the actinobacteria and proteobacteria with the exception of the α-proteobacterial ones. EcfM deduced orthologs were found among most proteobacterial sub-groups with the exception of *B. pseudomallei *and closely related species.

#### - *ecfF *and *ecfJ*: evidence of *Burkholderia*-specific lineages

EcfF and EcfJ are distinct deduced σ^70 ^factors which do not group on the NJ tree (Fig. 4) with any of the previously described ECF sigma factors available in the databases. They can thus be considered *Burkholderia*-specific. However, *ecfJ *was not detected in the genome of *B. xenovorans *LB400. The processes making use of these ECF factors remain unknown.

### Genomic rearrangements of σ^70 ^genetic loci among Bcc sequenced genomes

**Figure 5:**
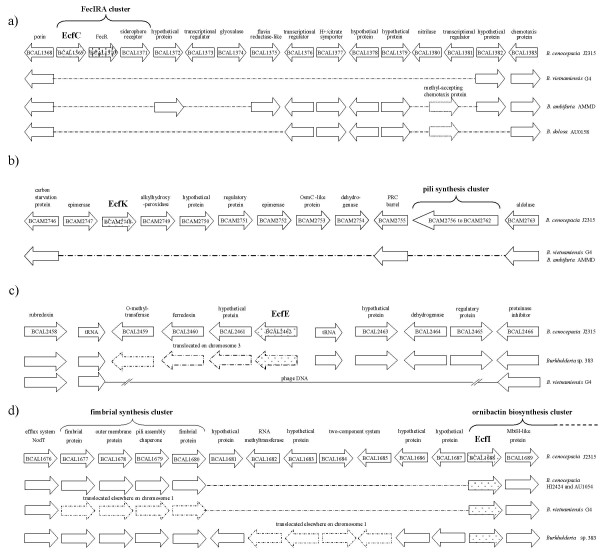
**Syntheny analysis of *B. cenocepacia *strain J2315 *ecf *genomic regions showing rearrangements in other Bcc sequenced genomes**. (a) *ecfC*, (b) *ecfK*, (c) *ecfE*, and (d) *ecfI *genomic regions. *Bcen*-J2315 concatenated genome was compared to other Bcc sequenced concatenated genomes using ACT [36]. *Bcen*-J2315 ORF annotations were assigned using other annotated *Burkholderia *genomes. ORF numbers assigned by the Sanger Institute are indicated. Dashes indicate missing regions. Phage DNA in (c) indicates a potential phage insertion. Arrows indicating ORF of sigma factors are filled with dots, and of anti-sigma factors are filled with vertical bars.

Inside the Bcc, syntheny analyses of the σ^70 ^genetic loci were performed using ACT [36]. Some of these results are shown in Fig. 5. *sigE, sigL, sigM, sigK, ecfA, ecfB, ecfD, ecfF, ecfJ, ecfL *and *ecfM *genetic loci were conserved over at least 10 kb (upstream and downstream) among the tested Bcc genomes. *ecfB *is located 5,5 kb upstream *ecfM*. *sigI*, *sigJ*, *ecfC*, *ecfF*, *ecfG*, *ecfH*, *ecfI*, and *ecfK *loci were affected by various genomic rearrangements. *ecfA, ecfB *and *ecfF *are physically linked to anti-sigma gene sequences. Three major categories of genomic rearrangementswere defined, one leading to a loss of a sigma gene through unknown processes, one leading to a loss of a sigma gene through integration of phage DNA, and one leading to a reorganization of the genetic environment surrounding the sigma gene.

#### - loss of σ^70 ^gene sequences in the Bcc: cases of *ecfC*, *ecfG*, *ecfH*, *ecfK*, *sigI*, and *sigJ *loci

In this category, in one instance, a very precise deletion of a sigma gene and its respective anti-sigma was observed. This situation was observed for *ecfG *and its anti-sigma in the *B. vietnamiensis *G4 genome. However, in most instances, the loss of a sigma gene was concomitant to the loss of important pieces of DNA. *sigI *and *sigJ *are, in fact, found deleted in respectively *Burkholderia *sp. 383, and *B. cenocepacia *PC184, and the extension of the deleted regions were respectively of 20 and 150 kb. Loss of *ecfH *in *B. vietnamiensis *G4 and *B. dolosa *AU0158 genomes were also associated with the deletion of a DNA region (9 and 12 kb, respectively). In the case of the *ecfK *locus, which is found at the proximity of a pilus synthesis cluster and genes involved in the synthesis of an alkylhydroperoxidase (Fig. 5b), its deletion involved a loss of about 7 kb in the genomes of *B. vietnamiensis *G4 and *B. ambifaria *AMMD. Interestingly, the configuration of these latter deletions were identical in these two genomes, suggesting that it had either occurred prior to the separation of these bacteria into two distinct species or involved similar processes. *ecfC *deletion event was observed among several genomes but the extent of the deletion events was variable. Nevertheless, one border was always conserved. *ecfC *(*fecI*-like) is deleted in *B. vietnamiensis *G4, *B. ambifaria *AMMD and *B. dolosa *AU0158 genomes (Fig. 5a). Part of the deletion concerned genes involved in iron-uptake. These genomic rearrangements of the *ecfC *genetic cluster could explain the differences observed between *B. cepacia *and *B. vietnamiensis *in response to iron starvation. *B. vietnamiensis *was only found to produce ornibactin under iron starvation while *B. cepacia *clinical isolates could also produce pyochelin and cepabactin [37]. Among the *B. ambifaria *and *B. dolosa ecfC *loci, 4 to 6 ORFs, over the eleven likely involved in iron-uptake in *Bcen*-J2315, were found conserved. One of these was found encoding a citrate symporter involved in the transport of Fe(III)-citrate complexes. To our knowledge, it is not yet known if *B. ambifaria *and *B. dolosa ecfC *loci are functional.

#### - loss of *ecfE *in *B. vietnamiensis*: involvement of phage DNA

Concerning this second category, syntheny analysis showed the *Bcen*-J2315 *ecfE *region to have been modified by the integration of phage DNA in *B. vietnamiensis *strain G4. In this genome, *ecfE *and an overall 5 kb region are replaced by phage DNA. All genes upstream the tRNA gene of the *ecfE *region are conserved. The loss of *ecfE *in *B. vietnamiensis *was confirmed by DNA blot analyses (Table 2). All restricted genomic DNA of the *B. vietnamiensis *strains tested did not show any DNA hybridization with an *ecfE *probe. Similarly, no positive hybrization signal could be obtained on *B. multivorans *DNA blots (Table 2), while positive signals were obtained for the other Bcc species. It is not known if the integration of phage DNA was involved in the deletion of *ecfE *among the *B. vietnamiensis *and *B. multivorans *strains analysed by DNA probings. However, we consider that this is highly probable.

#### - reorganized σ^70 ^genetic loci in the Bcc: cases of *ecfI *and *ecfE *loci

Concerning this third category, two cases were observed. One concerned the *ecfI *(*pvdS *homolog) region, and the other one the *ecfE *region. Regarding *ecfI *and its related genetic cluster, the ornibactin biosynthesis cluster, genes upstream *ecfI *are found deleted in *B. vietnamiensis *G4, *B. cenocepacia *HI2424 and AU1054 genomes, and translocated elsewhere in the *Burkholderia *sp. 383 genome. Genes involved in fimbrial synthesis, and the synthesis of a two component system were partly affected by these rearrangements (see Fig. 5d). Similarly, the *ecfE *region and three of its neighboring genes proximal to a tRNA gene sequence in *B. cenocepacia *J2315 chromosome 1 were translocated into a distinct region of the genome of *Burkholderia *sp. 383, while all other genes were kept in place (Fig. 5c).

## Conclusion

This paper was dedicated to the analysis of the σ^70 ^gene family, its architecture and plasticity, through an analysis of about 20 genome sequences of the eubacterial *Burkholderia *genus. Emphasis was made on a bacterial group of this genus which is named the "*Burkholderia cepacia *complex" or Bcc. Several epidemic clones have been described in the Bcc, including bacteria responsible of the "*cepacia *syndrome", a dramatic necrotizing pneumonia. The great versatility of these Bcc species could be linked to an important diversity in regulatory processes including those of the σ^70 ^gene family. The σ^70 ^gene family is widely distributed among the eubacteria, and probably emerged early in bacterial evolution. Duplication events led to the emergence of several Bcc σ^70 ^paralogs which were found limited to certain bacterial groups like the proteobacteria, *Burkholderia*, the *cepacia *complex, and particular Bcc species or strains. This gene family was found to be under strong selective pressures that could lead to acquisition/deletion (*ecfE*, *ecfG*, *sigI*, *sigJ*, *ecfC*, *ecfH*, and *ecfK*), and duplication events (*ecfA *and *rpoD*) modifying its architecture. These changes are likely to be key events in Bcc evolution, generating novel gene expression profiles that could be more suited for the colonization of particular biotopes including the respiratory tract of CF patients. A key conclusion was that the comparative analysis of the σ^70 ^gene architecture of Bcc species and of *Pseudomonas aeruginosa*, a species found in similar habitats including CF lungs, revealed distinct evolutionary strategy, with the Bcc having selected several alternative primary factors, something not recorded among *P. aeruginosa *and only reported to occur among the actinobacteria. Only three ECF paralogs (FecI-like, PvdS and AlgU) were found in common between the Bcc and *P. aeruginosa*. The next step in these studies will be to investigate the impact of gains and losses of σ^70 ^determinants in the ecology of the Bcc and the colonization of the CF lungs.

## Methods

### *B. cenocepacia *genome sequence, and σ^70 ^BLAST and ORF searches

TBLASTN searches of *B. cenocepacia *J2315 genome were performed using the Wellcome Trust Sanger Institute web site [26]. The following ten σ^70 ^factors were used for these searches: RpoD (accession #AAA24601), RpoS (#CAA34435), RpoH (#AAA24587), SigF (#AAA22788), FliA (#BAA00389), AlgU (#AAC43714), CnrH (#AAA21967), HrpL (#AAD00805), PpuI (#CAA54870), and RfaY (#AAA92044). All loci encoding putative proteins showing good similarities with the target proteins were analysed by FramePlot [38], ORF finder [39] and testcode [40]. BLASTP analyses of the deduced ORF proteins were performed [41]. Conserved domains among sigma factors were detected using CDD 2.02 [42]. To achieve saturation in the detection of σ^70 ^factors, identified putative factors were concatenated and the resulting sequence compared to *Bcen*-J2315 chromosomes using ACT. This graphical tool allows visualisation of a BLAST comparison of two sequences. A TBLASTN was performed using *Bcen*-J2315 chromosomes as the database and the concatenated sigma sequences as the query. Visual identification of DNA regions showing similarity with different sigma factors was performed and candidate ORF were analysed as stated above.

### Molecular phylogenetic analyses

Multiple alignments were computed using CLUSTALW [43]. Distances between sequence pairs, the inferred phylogenetic trees, and bootstrap values were all computed through the Mega3 graphic tool [44]. Phylogenetic trees were built using the Neighbor-Joining (NJ) method [45]. Bootstrap replicates were performed using 1000 replicates. Gap containing sites were deleted globally or by pair of sequences. Alignments can be downloaded using the UMR5557 http server [46,47].

### Bacterial strains, DNA extractions, PCR amplification and DNA blot hybridizations

Strains used in this study are listed in Table 2. Bacteria were grown in LB (Luria-Bertani) [48] broth at 30°C. DNA extractions were performed as previously described [49]. PCR amplifications (25 μl) were done according to the *Taq *polymerase manufacturer (Invitrogen, Cergy Pontoise France) without 1% w-1 (detergent) but 1,25 μL of DMSO. PCR primers are indicated in Table 3. PCR cycles using total DNA were as follow: (1) 95°C for 5 min, (2) 95°C for 30 sec, selected annealing temperature for 30 sec (see Table 3), 72°C for 30 sec (35 cycles), (3) 72°C for 5 min.

Genomic DNA blots were performed on Genescreen (USA) nylon membranes following [50]. The DNA (5 μg) was digested with *Eco*RI (Fermentas) at 1 U/μg DNA, for 16 h at 37°C. DNA probes were labelled using the random priming labelling kit (Amersham-Pharmacia, Orsay-France). DNA hybridizations were carried out according to the nylon membrane manufacturer (Genescreen, USA). Membranes were hybridized and washed at 65°C. Autoradiography was performed according to [50].

## Authors' contributions

AM performed computer and molecular analyses, had significant inputs in the conception of this work, and drafted parts of the manuscript. PEDLS and AG helped performed computer and molecular analyses, and made substantial contributions in the acquisition of data. BC conceived the study, designed the experiments, participated in most analyses, coordinated the work, and drafted the manuscript. All authors read and approved the final manuscript.
